# Anticancer Activity of Rutin and Its Combination with Ionic Liquids on Renal Cells

**DOI:** 10.3390/biom10020233

**Published:** 2020-02-04

**Authors:** Rita Caparica, Ana Júlio, Maria Eduarda Machado Araújo, André Rolim Baby, Pedro Fonte, João Guilherme Costa, Tânia Santos de Almeida

**Affiliations:** 1 CBIOS—Universidade Lusófona’s Research Center for Biosciences & Health Technologies, Campo Grande 376, 1749-024 Lisboa, Portugal; rita.caparica@ulusofona.pt (R.C.); ana.julio@ulusofona.pt (A.J.); prfonte@ualg.pt (P.F.); 2 Department of Biomedical Sciences, University of Alcalá, Ctra. Madrid-Barcelona Km. 33.600, Alcalá de Henares, 28871 Madrid, Spain; 3 CQE, and Department of Chemistry and Biochemistry, Faculty of Sciences, University of Lisbon, Campo Grande 1749-016 Lisboa, Portugal; mearaujo@fc.ul.pt; 4 Department of Pharmacy, School of Pharmaceutical Sciences, University of São Paulo, 580 Prof. Lineu Prestes Av., Bl. 15, São Paulo, SP 05508-900, Brazil; andrerb@usp.br; 5 iBB-Institute for Bioengineering and Biosciences, Department of Bioengineering, Instituto Superior Técnico, Universidade de Lisboa, 1049-001 Lisboa, Portugal; 6 Center for Marine Sciences (CCMar), University of Algarve and Department of Chemistry and Pharmacy, 8005-139 Faro, Portugal

**Keywords:** rutin, renal cancer, 786-O cells, Vero cells, cytotoxicity, cell cycle, ionic liquids, Solubility, ILs–nanoparticles hybrid system

## Abstract

The renal cell carcinoma (RCC) is the most common type of kidney cancer. Identifying novel and more effective therapies, while minimizing toxicity, continues to be fundamental in curtailing RCC. Rutin, a bioflavonoid widely found in nature, has shown promising anticancer properties, but with limited applicability due to its poor water solubility and pharmacokinetics. Thus, the potential anticancer effects of rutin toward a human renal cancer cell line (786-O), while considering its safety in Vero kidney cells, was assessed, as well as the applicability of ionic liquids (ILs) to improve drug delivery. Rutin (up to 50 µM) did not show relevant cytotoxic effects in Vero cells. However, in 786-O cells, a significant decrease in cell viability was already observed at 50 µM. Moreover, exposure to rutin caused a significant increase in the sub-G1 population of 786-O cells, reinforcing the possible anticancer activity of this biomolecule. Two choline-amino acid ILs, at non-toxic concentrations, enhanced rutin’s solubility/loading while allowing the maintenance of rutin’s anticancer effects. Globally, our findings suggest that rutin may have a beneficial impact against RCC and that its combination with ILs ensures that this poorly soluble drug is successfully incorporated into ILs–nanoparticles hybrid systems, allowing controlled drug delivery.

## 1. Introduction

Renal cell carcinoma (RCC) is the most common type of kidney malignancy in adults that is established in the renal proximal convoluted tubules [1,2]. RCC is highly vascularized and can metastasize to different body sites, including lungs, liver, and bones [2,3]. There are different histological subtypes of RCC [1]. Among them, the clear cell RCC (ccRCC) is the most common type, comprising approximately 70%–80% of the RCC cases [3,4,5,6]. The incidence of RCC varies in different countries and also with age, gender, and race. 

Regarding the treatment, refractoriness and low response rates to adjuvant therapies, such as radiotherapy, targeted therapy, chemotherapy, hormonal therapy, and immunotherapy, are clear limitations [3,4,5]. Thus, surgery is the preferred treatment for localized primary tumors. However, the effectiveness of the surgery highly depends on the grade and stage of the disease and the presence or absence of metastasis [4,5]. Therefore, it becomes essential to find new, safe, and more effective treatments against RCC.

Natural compounds have been important tools for research and development of new drugs [7,8,9,10]. Among them, flavonoids, which are polyphenolic compounds that occur naturally in plants, have emerged as promising resources to prevent and treat human diseases, including cancer, due to their biological properties [7,11,12]. 

Rutin is a representative flavonoid widely found in natural sources, including fruits (e.g., apples, grapes, lemons), vegetables (e.g., carrots, potatoes) and beverages (e.g., tea and wine) [13,14,15,16], that has been highlighted for its therapeutic potential [17]. Many studies have demonstrated several pharmacological properties of rutin, including anti-inflammatory, antioxidant, antidiabetic, vasoprotective, antimicrobial, and anticancer activities [13,15,16]. More specifically, previous studies showed that rutin is able to reduce tumor growth and induce cell cycle arrest and apoptosis in several in vitro cell models [18,19,20]. Despite the pharmacological potential of this bionatural compound, its applicability remains a major concern since rutin is not able to be absorbed in its native form; instead, it is hydrolyzed into quercetin, which is then absorbed and presented as conjugated metabolites in the circulation [21,22]. Furthermore, the low solubility of rutin impairs its bioavailability and formulation into delivery systems [22,23]. 

Several strategies have been used to increase the solubility of poorly soluble drugs [23,24,25]. Over the last few years, some studies have shown that ionic liquids (ILs) have a positive impact on increasing the solubility of drugs [24,26,27,28]. More recently, a study conducted by our group demonstrated that choline-based ILs improved the solubility of poorly water-soluble compounds and allowed their incorporation, in higher amounts, into O/W emulsions [23]. Thus, ILs may act as functional excipients and allow not only the use of poorly soluble drugs but may also be valuable in the development of new and more efficient delivery systems. 

ILs are organic salts that have melting points below 100 °C or, in some cases, are liquid at room temperature. These salts have been used in several areas due to their numerous and remarkable properties such as their low vapor pressures, high thermal and chemical stability, the ability to dissolve organic, inorganic, and polymeric materials and the fact that they can be modified and their properties modulated through synthesis [23,24,29,30,31,32,33,34]. Nonetheless, the toxicity of these salts may be quite variable and to prove their safety and usefulness at non-toxic concentrations, toxicity studies should always be considered. In this context, choline-based ILs have shown promising properties as functional excipients [23,24,26,27], namely, as drug solubility/loading enhancers. 

Bearing all of this in mind, the aim of the present work is to study the potential anticancer activity of rutin towards a human renal cancer cell line while considering its impact on normal kidney cells. Due to the low solubility of rutin and considering the valuable characteristics of choline-amino acid ILs, these salts were used to enhance the solubility of rutin. Additionally, considering the low bioavailability and the potential applicability of rutin, hybrid IL-nanocarriers, containing this bioactive compound and ILs, were prepared and their physicochemical properties and drug release profile were evaluated. Furthermore, the impact of rutin individually, and in combination with each IL, on both renal cell models was also assessed.

## 2. Materials and Methods

### 2.1. Chemicals

The reagents and solvents used for the synthesis of the ILs were choline hydroxide in methanol [Cho][OH]/MeOH 45% and methanol, both from Sigma-Aldrich (Saint Louis, MO, USA), also acetonitrile from VWR (Fontenay-sous-Bois, France) and the amino acids, L-phenylalanine and glycine, from PanReact AppliChem (Barcelona, Spain).

For the cytotoxicity studies, the following reagents were purchased from Sigma-Aldrich (Saint Louis, MO, USA), phosphate buffered saline (PBS; 0.01 M, pH 7.4), trypsin, penicillin–streptomycin (pen/strep) solution, thiazolyl blue tetrazolium bromide (MTT) and dimethyl sulfoxide (DMSO). Fetal bovine serum (FBS) and Dulbecco’s Modified Eagle’s Medium (DMEM) were from Biowest (Nuaillé, France). The propidium iodide (PI) was purchased from Merck (Darmstadt, Germany). All the rutin solutions had a final concentration of 0.5% (*v/v*) and were prepared in DMSO, for all assays. The solutions containing ionic liquids were all prepared in sterile water.

For the production of the nanoparticles, the dichloromethane and the polyvinyl alcohol (PVA) were obtained from Sigma-Aldrich (St. Louis, MO, USA). Corbion Purac (Amsterdam, The Netherlands) kindly supplied the Poly(lactic-co-glycolic acid) (PLGA) 50:50 with acidic termination (Purasorb^®^ PDLG 5002A). 

Rutin was obtained from Fagron, São Paulo, Brazil. 

### 2.2. Ionic Liquids Synthesis

Two choline-amino acids ILs, the 2-hydroxyethyl-trimethylammonium L-phenylalaninate [Cho][Phe] and the 2-hydroxyethyl-trimethylammonium glycinate [Cho][Gly] were synthesized according to a previously described procedure [24]. The obtained ILs were characterized by ^1^H NMR and ^13^C NMR spectra using a Brucker Avance 400^®^ apparatus (Billerica, MA, USA), at 400 MHz, in D_2_O.

### 2.3. Cell Culture

In this study, the Vero-E6 normal kidney cells (ATCC^®^ CRL-1586™), and the 786-O human renal cancer cells (ATCC^®^ CRL-1932™), were obtained from the American Type Culture Collection (ATCC; Manassas, VA, USA). The Vero-E6 and 786-O cells were cultured in low and high glucose DMEM medium, respectively, supplemented with 10% FBS and 1% pen/strep. Cells were maintained at 37 °C in a humidified air atmosphere containing 5% CO_2_. 

### 2.4. MTT Assay

To evaluate the cytotoxicity of rutin and of the ILs, individually and in combination, cell viability was determined using the MTT reduction assay. Vero and 786-O cells were seeded at a density of 5 × 10^3^ and 3 × 10^3^ per well, respectively, in 200 μL culture medium in 96-well plates and incubated for 24 h. Cells were then incubated, either with rutin (0–250 μM), with [Cho][Phe] (0–0.5%, *v/v*) or with [Cho][Gly] (0–0.5%, *v/v*) individually, or, with rutin (0–250 μM) in combination with [Cho][Phe] (0.3%, *v/v*) or with [Cho][Gly] (0.2%, *v/v*) for 48 h. The MTT reduction assay was then carried out according to previously published procedures [24,35]. Absorbance values for untreated control cells correspond to 100% of cell viability. For this assay, two to seven independent experiments were carried out and at least four replicate cultures were used in each independent experiment.

The half-maximal inhibitory concentration (IC_50_) was calculated using the GraphPad Prism 7^®^ Statistical Software (San Diego, CA, USA).

### 2.5. Solubility Studies

The solubility studies were performed according to a previously published procedure [23]. Briefly, several saturated solutions of rutin were prepared in triplicate, in water and water:IL mixtures, containing 0.3% of [Cho][Phe] or 0.2% of [Cho][Gly] IL. Then, in a horizontal shaker (IKA VIBRAX VXR^®^, LTF Labortechnik GmbH & Co., Bodensee, Germany), the solutions were shaken during 72 h at 25 °C. Next, all solutions were filtrated and analyzed using an UV–visible spectrophotometry Evolution^®^ 300 from Thermo Scientific (Hertfordshire, England) at 353 nm, the maximum absorption wavelength of rutin in water.

### 2.6. Flow Cytometric Analysis of DNA Cell Cycle

The cell cycle distribution of cells treated with rutin and ILs, either individually or in combination, was determined by flow cytometry. This assay was carried out according to previously published procedures [6,36]. Briefly, for cell-cycle analysis, 786-O cells were seeded at a density of approximately 5 × 10^4^ per well in 6-well plates and cultured for 24 h. Afterwards, rutin (50 µM), [Cho][Phe] (0.3%, *v/v*) or [Cho][Gly] (0.2%, *v/v*) were added and cells were incubated for a further 48 h period. Cells were then harvested using EDTA (5 mM) in PBS, washed with cold PBS and fixed with 80% ethanol. Then, the cells were treated with RNase A (20 μg/mL) and stained with propidium iodide (10 μg/mL) for 20 min and analyzed using a FACSCalibur flow cytometer (BD Biosciences, Franklin Lakes, NJ, USA). The data acquisition and analysis were executed using CellQuest software (Becton Dickinson, San Jose, CA, USA) and FlowJo (Tree Star, San Carlos, CA, USA), respectively. Three independent experiments were executed.

### 2.7. Production of the IL-Nanoparticles Hybrid System

The IL-nanoparticle hybrid systems were prepared using a water-in-oil-in-water (W/O/W) double emulsion method, according to previously published procedures [37]. Briefly, an aqueous solution of rutin with IL was produced using the highest concentration of drug dissolved by each IL (2.49 mg/mL for [Cho][Phe] and 1.73 mg/mL for [Cho][Gly]). The used IL percentage was 0.3% *(v/v)* for [Cho][Phe] and 0.2% *(v/v)* for [Cho][Gly], which is the IL concentration where Vero cell viability was maintained. Then, 200 µL of the rutin:IL solution was poured into 200 mg PLGA dissolved in 2 mL of dichloromethane. This mixture was sonicated for 30 s at 70% of amplitude using a Q125 Sonicator (QSonica Sonicators, Newtown, CT, USA), obtaining the first emulsion. The latter was then poured into 25 mL of a PVA 2% (*w/v*) solution and sonicated at the same previous conditions, obtaining the secondary emulsion. The formulation was then placed under magnetic stirring until evaporation of the organic solvent. All formulations were prepared in triplicate.

### 2.8. Particle Size, Polydispersity Index and Zeta Potential Analysis

The IL-nanoparticle hybrid system was characterized in terms of its polydispersity index (PdI) and particle size by dynamic light scattering using the Delsa™ Nano C (Beckman Coulter, Inc., Brea, CA, USA) and zeta potential by the electrophoretic mobility analysis using Malvern^®^ NanoSizer (Worcestershire, United Kingdom). All samples were prepared in triplicate and analyzed at 23 ± 2 °C. 

### 2.9. Association Efficiency and Loading Capacity of Rutin

For the evaluation of the association efficiency (AE) and loading capacity (LC) of rutin, all formulations were centrifuged at 16,350× *g* for 15 min at 4 °C and then the supernatant was collected. Through UV spectroscopy, rutin was quantified in the supernatant at 353 nm (maximum absorption wavelength in the PVA solution). The pellet resuspended in water and freeze-dried in a LABCONCO FreeZone 25^®^ freeze dryer (Kansas City, MO, USA) at 400 mTorr for 24 h and −50 °C of condenser surface temperature.

The AE of rutin was determined using Equation (1):

(1) $$\mathrm{AE}=\frac{\mathrm{Total}\;\mathrm{amount}\;\mathrm{of}\;\mathrm{rutin}-\mathrm{Free}\;\mathrm{amount}\;\mathrm{of}\;\mathrm{rutin}\;\mathrm{in}\;\mathrm{the}\;\mathrm{supernatant}}{\mathrm{Total}\;\mathrm{amount}\;\mathrm{of}\;\mathrm{rutin}}\times 100$$

Additionally, for determining the LC of rutin, Equation (2) was used:

(2) $$\mathrm{LC}\;=\frac{\mathrm{Total}\;\mathrm{amount}\;\mathrm{of}\;\mathrm{rutin}-\mathrm{Free}\;\mathrm{amount}\;\mathrm{of}\;\mathrm{rutin}\;\mathrm{in}\;\mathrm{the}\;\mathrm{supernatant}}{\mathrm{Total}\;\mathrm{dry}\;\mathrm{mass}\;\mathrm{of}\;\mathrm{nanoparticles}}\times 100$$

### 2.10. In Vitro Release Study

For the release study, the nanoparticle suspension was centrifuged at 12,600× *g* for 20 min at 4 °C. The supernatant was removed, and the pellet was resuspended in 10.0 mL of a PBS solution. Then, the solutions were incubated at 37 °C and stirred at 100 rpm in a Heidolph^®^ 1000 incubator with motor Heidolph^®^ Unimax 1010 (Schwabach, Germany). Next, at predetermined time intervals (30 min, 1, 2, 4, 6, 8, 12, 24, 48, and 72 h), aliquots of each sample (1 mL) were taken and replaced by the same volume of PBS. The samples were centrifuged at 12,600× *g* for 15 min at 25 °C and the drug present in the supernatant was quantified at 353 nm in the UV–visible Spectrophotometer Evolution^®^ 300 from Thermo Scientific (Hertfordshire, England).

### 2.11. Statistical Analyses

Differences in mean values of the results were evaluated with one-way analysis of variance (ANOVA) and then followed by Tukey’s multiple comparison test, after assessing normality. The analyses were performed with SPSS statistical package (version 25, SPSS Inc. Chicago, IL) and GraphPad Prism 7^®^ from GraphPad Software (San Diego, CA, USA).

## 3. Results

### 3.1. Synthesis of ILs

Both prepared choline-amino acid ILs, [Cho][Phe] and [Cho][Gly], revealed to be viscous at room temperature and their structures were confirmed by ^1^H NMR and ^13^C NMR and the obtained results are in agreement with the literature [23,34].

### 3.2. Cell Viability of Renal Cells

#### 3.2.1. Effect of Rutin on the Viability of Renal Cells

In the present study, the MTT assay was used to evaluate the impact of rutin (0–250 µM; 48 h) treatment on the cell viability of two renal cell lines, the Vero normal kidney cells, and the 786-O human renal cancer cells. 

**Figure 1:**
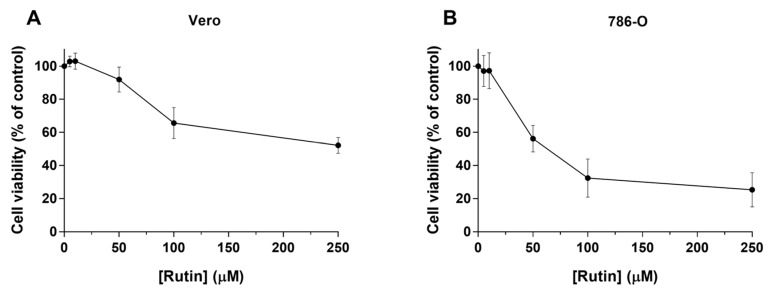
Cytotoxic effects of rutin (0–250 µM; 48 h) in Vero (**A**) and 786-O (**B**) cells. The viability of rutin-exposed cells was evaluated by MTT assay. Values represent mean ± SD (*n* = 6–7) and are expressed as percentages of the non-treated control cells.

The results with Vero cells only showed a significant decrease in cell viability at the two highest studied concentrations of rutin (100 and 250 µM), with the respective viabilities being 65.6% and 52.1% (Figure 1A).

On the other hand, for the 786-O cancer cells, a concentration-dependent decrease of cell viability at much lower concentrations than in Vero cells was observed, with an IC_50_ value of 45.2 µM (Figure 1B). The results showed that rutin induced a remarkable decrease in cell viability, compared with non-treated control cells at concentrations of 50, 100, and 250 µM, reaching 56.1%, 32.4%, and 25.3%, respectively.

#### 3.2.2. Effect of the Two Choline-amino Acid ILs on the Viability of Renal Cells

**Figure 2:**
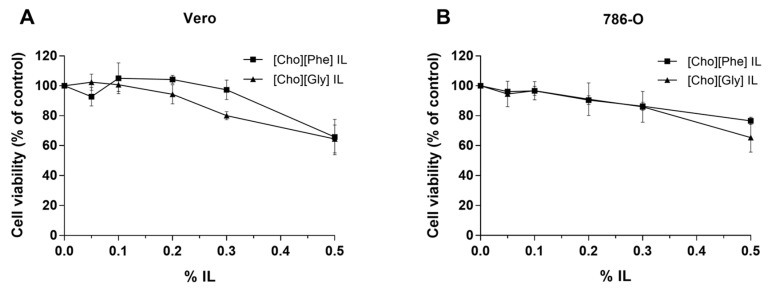
Cell viability of Vero (**A**) and 786-O (**B**) cells exposed to choline-amino acid ILs (0%–0.5%, *v/v*). The cell viability of ILs-exposed cells (48 h) was evaluated by MTT assay. Values represent mean ± SD (*n* = 2–5) and are expressed as percentages of the non-treated control cells.

Since ILs may be used as functional excipients to allow the incorporation of rutin in delivery systems, it was also relevant to understand their influence on cell viability. Hence, the effect of the two prepared choline-amino acid ILs (0%–0.5% (*v/v*); 48 h) on Vero and 786-O cells viability was evaluated using the MTT assay. The exposure to the [Cho][Phe] and [Cho][Gly] ILs induced a concentration-dependent decrease of cell viability on both renal cell lines (Figure 2). Furthermore, the results also showed that at concentrations up to 0.3% of [Cho][Phe] and up to 0.2% of [Cho][Gly], the cell viability was maintained in both cell lines. 

#### 3.2.3. Impact of the Co-treatment of Rutin with ILs on the Viability of Renal Cells

**Figure 3:**
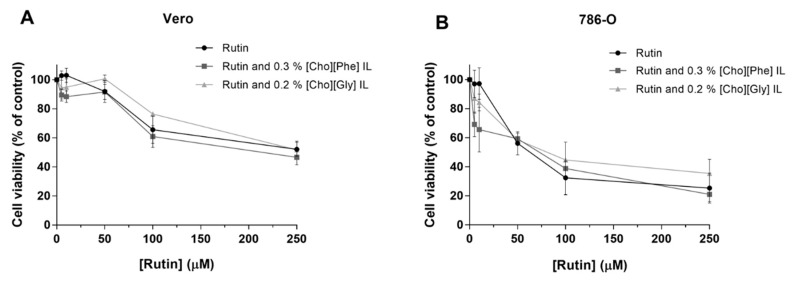
Cytotoxic effects of rutin (0–250 µM) individually and in combination with 0.3% of [Cho][Phe] or 0.2% of [Cho][Gly] ILs in Vero (**A**) and 786-O (**B**) cells. The cell viability was evaluated by MTT assay (48 h). Values represent mean ± SD (*n* = 3–7) and are expressed as percentages of the non-treated control cells.

It was also important not only to evaluate the effect of rutin and ILs individually but also of the combined treatment of rutin (0–250 µM) with each IL (0.3% of [Cho][Phe] or 0.2% [Cho][Gly] (*v/v*)), using the same experimental conditions. In this case, the results showed that in general, the presence of each IL did not lead to significant differences in the cell viability when compared with the viability of rutin-treated cells in both cell lines (Figure 3). Subsequently, considering all the results up to this point, for the following studies, the concentration of 50 µM for rutin and the concentrations of 0.3% for [Cho][Phe] and 0.2% for [Cho][Gly] were selected.

### 3.3. Cell Cycle Distribution of 786-O Cells Treated with Rutin Individually and in Combination with ILs

The influence of the individual treatment with rutin or ILs and the combination of rutin with ILs in the human renal cancer cell cycle progression was studied by assessing the cellular DNA content.

**Figure 4:**
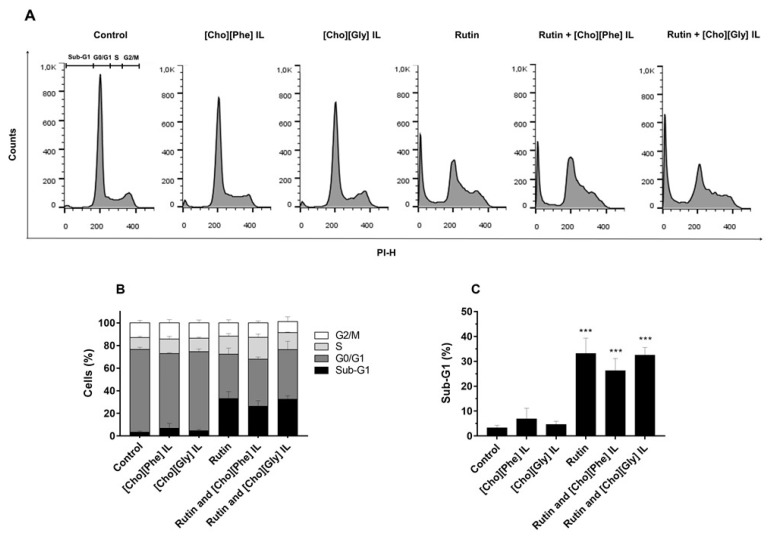
Effect of rutin and ILs, individually and combined, on cell cycle progression of 786-O cells. Cellular DNA content was analyzed by flow cytometry after 48 h incubation with rutin (50 µM) and/or each IL, [Cho][Phe] IL (0.3%) or [Cho][Gly] IL (0.2%). Representative flow cytometry histograms (**A**), Sub-G1, G0/G1, S and G2/M populations summary results (**B**) and Sub-G1 population percentage (**C**). Values represent mean ± SD (*n* = 3–6); *** *p* < 0.001 (one-way ANOVA, Tukey’s test, relative to control cells).

The exposure of 786-O cells to [Cho][Phe] or [Cho][Gly] ILs (0.3% and 0.2%, respectively; for 48 h) led to a cell cycle distribution similar to that of control cells (Figure 4). The exposure to rutin (50 µM; 48 h) caused a significant increase of approximately 30% in the sub-G1 population (*p* ˂ 0.001), an increase in the S population, and a decrease in G0/G1 population of 786-O cells when compared with the untreated control cells. Regarding the combination of rutin (50 µM) with [Cho][Phe] or [Cho][Gly] ILs (0.3% and 0.2%, respectively), no relevant and statistical differences were observed when compared to rutin-exposed cells (Figure 4). 

### 3.4. Solubility of Rutin in the Presence of Choline-amino Acid ILs

The impact of the two choline-amino acid ILs on the solubility of rutin was investigated through solubility studies in aqueous solutions in the presence of 0.3% of [Cho][Phe] IL or 0.2% of [Cho][Gly] IL. Results showed that the solubility of rutin was enhanced from 0.19 mg/mL, in water, to 2.49 mg/mL when using [Cho][Phe] IL and to 1.73 mg/mL in the presence of [Cho][Gly] IL (Table 1). 

### 3.5. Ionic Liquids-Nanoparticles Hybrid Systems Containing Rutin

#### 3.5.1. Particle Size, Polydispersity Index and Zeta Potential Analysis

**Figure 5:**
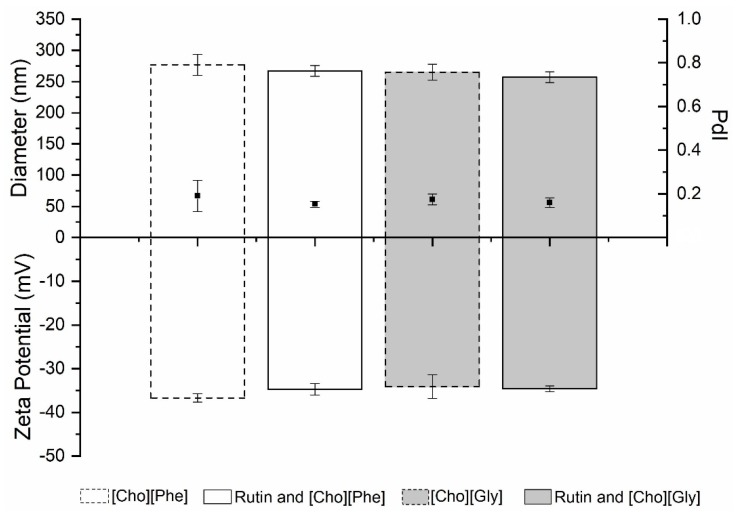
Diameter (nm) (top bars), PdI (black squares) and zeta potential (mV) (bottom bars) of unloaded and rutin-loaded IL-PLGA nanoparticle hybrid systems (*n* = 3, mean ± SD).

Regarding the physicochemical proprieties, the IL-nanoparticle hybrid systems showed a diameter ranging between 250–300 nm, with a PdI around 0.2 and a zeta potential of about −35 mV (Figure 5). No relevant differences were found on these parameters between loaded and unloaded delivery systems. Also, the results obtained for the formulations with the two different ILs were very similar.

#### 3.5.2. Association Efficiency (AE) and Loading Capacity (LC) of Rutin

The AE and the LC of the rutin loaded into the nanoparticles were also evaluated. The AE of rutin in the delivery systems were 84.5% ± 0.3%, in the presence of [Cho][Phe], and 84.7% ± 0.3%, with [Cho][Gly] (Table 2). The LC values were also similar for both formulations (Table 2). All results showed that there are no significant differences in the AE and LC between both ILs.

#### 3.5.3. In Vitro Release Study

**Figure 6:**
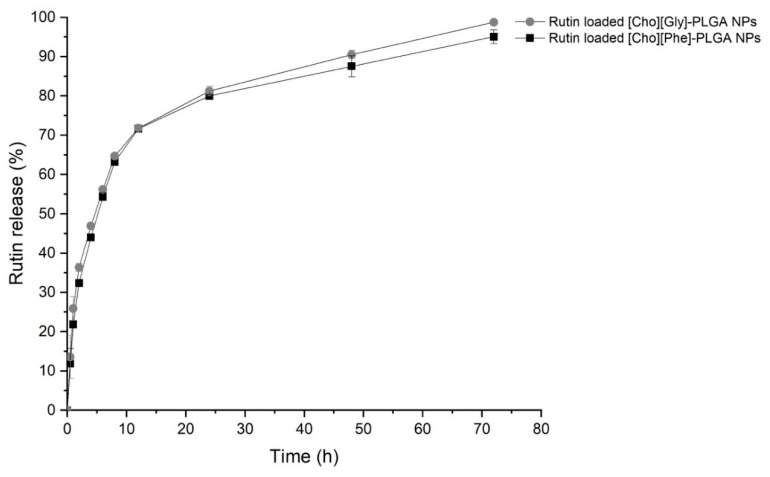
Cumulative release profile of the rutin-loaded IL-PLGA nanoparticle hybrid systems for 72 h in phosphate buffer saline at pH 7.4. Data represented as mean ± SD (*n* = 3). Statistical analysis performed with one-way ANOVA, Tukey’s test.

Concerning the in vitro release study, the release profile shows an initial burst in the first 5 h of the study (Figure 6). After that, the IL-nanoparticles hybrid systems showed a sustained drug release over time (Figure 6), with a percentage of rutin released ranging from 95.0% to 98.0%, in the presence of [Cho][Phe] or [Cho][Gly], respectively, after 72 h (Figure 6), with no significant differences between both choline-amino acid ILs.

## 4. Discussion

The ccRCC represents the most common subtype of RCC, comprising about 75% of all RCC tumors [6]. The mechanism that regulates RCC growth remains unclear. Moreover, the low sensitivity to conventional therapies and the low response rate to targeted therapies limits the treatment of this type of renal cancer [1,4,5,38]. Thus, the discovery of new and/or more effective and safe therapies becomes essential. In previous studies, rutin has shown biological activity toward different cancer cell lines, indicating that this natural compound could be a promising anticancer agent [8,16,18,19,20,39]. However, despite its potential anticancer activity, its low solubility represents a challenge and limits the applicability of rutin. Bearing this in mind, the present study aimed to evaluated the impact of rutin on 786-O cells, a well-established and characterized human renal cancer cell line [6] and the applicability of ILs to improve the solubility and delivery of this biomolecule, while considering the safety and applicability of this natural compound alone and when combined with the ILs. 

Herein, the impact of rutin (0–250 µM; 48 h) treatment on the cell viability of two renal cell lines, the Vero normal cells and the 786-O cancer cells, was obtained using the MTT assay. It was important to evaluate the effect of rutin on the viability of a representative kidney cell model, the Vero cells [40], to understand if this compound can be used safely. Our results showed that rutin did not induce relevant cytotoxicity up to 50 µM, although at concentrations higher than 50 µM the cell viability of Vero cells significantly decreased. 

Furthermore, to understand the anticancer potential of this natural compound, a concentration-response curve of rutin in 786-O human renal cancer cells was established. It was observed a clear concentration-dependent cytotoxicity effect of rutin, with a remarkable decrease at 50-µM concentration. These results are in agreement with previously published data performed in the same or different experimental conditions but using different cancer cell lines [18,39,41]. Moreover, with this study, it was possible to demonstrate that in the presence of rutin, 786-O cells are more sensitive to its cytotoxic effects than other previously studied cancer cell lines, namely, hepatoma cells of *Rattus novergicus* (HTC), human breast cancer cells (MDA-MB-231 and MCF-7), and colon cancer cells (HT29 and Caco-2) [7,9,23,39].

Hence, it is relevant to point out that these results reveal that rutin exhibits a higher cytotoxic effect against the 786-O cancer cells than against the non-cancer Vero cells, showing the potential anticancer activity of rutin against RCC. In addition, the results show that in further studies, 50 µM should be the upper concentration of rutin used to ensure both efficacy and safety.

Despite these promising results, rutin has very low solubility in water, 0.2 mg/mL [23], which limits its incorporation in delivery systems and, consequently, its applicability. Recently, a previous study from our group showed that the presence of ionic liquids, at non-toxic concentrations, enhanced the solubility of rutin and its loading into O/W emulsions, thus indicating that these salts may be used as green functional excipients [23]. However, since in this previous study the experimental conditions, the cell model and one of the ILs used were different ([Cho][Gly]), it becomes relevant for the present study to assess, for the first time, the influence of [Cho][Phe] and [Cho][Gly] ILs on the cell viability of the renal cell lines under study. Our results showed that both ILs induced a concentration-dependent decrease in cell viability in both cell lines. This may be due to the fact that the choline-based cation is a quaternary ammonium compound and these compounds are known cationic surfactants [42], which may justify the decrease in cell viability. Nonetheless, this cytotoxicity is not as pronounced as for ILs containing alkyl side chains, such as imidazole-based ionic liquids [24]. Furthermore, and even though, the toxicity of ILs has been mostly attributed to the cation, particularly the type of cationic head group and the length of the alkyl side chain, it is also known that the type of anion may have some impact on this toxicity [43], which justifies the observed differences between the two studied ILs. In fact, it has been shown that different amino acids lead to different cytotoxicities, with the elongation of the amino acid side chain being one of the structural contributions to enhance their cytotoxicity, although the toxicity sequence may differ depending on the model system used [43]. Moreover, our results also revealed that in further studies, to ensure safety, the highest concentration of ILs used should be 0.3% (*v/v*) for [Cho][Phe] and 0.2% (*v/v*) for [Cho][Gly] since up to these concentrations no significant cytotoxic effects were observed in Vero cells. Then, it was crucial to assess the solubility of rutin in the presence and absence of the studied ILs. Previous reports have shown that choline-amino acid ILs are able to enhance drug solubility [23,24,27]. For instance, our group has shown that the choline-amino acid IL, [Cho][Phe] and another choline-amino acid IL, [Cho][Glu], both increased the solubility of ferulic acid and rutin. Following our previous results, in the present study, we considered for the first time the impact of the studied ILs on the viability of a normal renal cell line (Vero), to ensure the functionality of these ILs, as solubility promotors of rutin, to evaluate their combination for possible anticancer applicability. To achieve this, we assessed if at non-toxic concentrations, in the normal renal cells, the ILs have a significant impact on rutin solubility. This functionality was in fact shown since the presence of each IL allowed a 9-fold and 13-fold increase on rutin solubility in the presence of 0.2% of [Cho][Gly] and 0.3% of [Cho][Phe], respectively, which is a considerable enhancement. 

To recap, at this point, our results allowed us to select for following studies the concentration of 50 µM of rutin and the concentrations of 0.3% (*v/v*) for [Cho][Phe], or 0.2% (*v/v*) for [Cho][Gly], since at these concentrations, the significant cytotoxic effect of rutin was only observed in 786-O cells and not in the normal Vero cells and also because the ILs considerably enhance the solubility of rutin at the chosen concentrations. 

Hereafter, to further explore the potential applicability of rutin combined with ILs, it is crucial to assess not only the individual effects of rutin, and each IL but also to evaluate the impact of the combined treatment with rutin and each IL on the cytotoxicity. The obtained results were very promising since, at the chosen concentrations of rutin and each IL, no significant differences were observed between the rutin-exposed cells and the cells exposed to the combination of rutin with each IL. This shows the functionality of the ILs since at biocompatible concentrations, their presence allows the maintenance of the rutin’s activity while allowing a higher drug solubility and delivery. 

Drug-induced cytotoxicity is frequently accompanied by cell cycle modification. Thus, after the selected concentrations from the cell viability studies, the impact of rutin on the human renal cancer cell cycle was also assessed to continue to evaluate the anticancer effects of this natural product.

In this study, rutin showed an impact on the cell progression of 786-O cells, leading to a significant increase in the sub-G1 population, with a consequent decrease in the G0/G1 population. This is in accordance with the previous results obtained for the cell viability, which reinforces the possible anticancer activity of this biomolecule. 

The rutin’s anticancer mechanisms of action are not yet fully understand. However, besides the impact of rutin on cell viability and cell cycle, shown herein, there are also some studies in other cancer cell models showing that this compound interferes with some carcinogenesis mechanisms, such as chemotactic ability [18] and cell adhesion and migration [39]. Additionally, rutin could also reverse multidrug resistance by the inhibition of P-glycoprotein [9]. 

Despite the fact that rutin’s mode of action is not totally comprehended, this study clearly suggests the possible applicability of rutin for renal cancer treatment. Furthermore, considering this applicability, the encapsulation in nanoparticles could be a relevant strategy to enhance therapeutic efficiency [44]. Nonetheless, the low aqueous solubility of rutin represents an impairment for this incorporation in drug delivery systems. In fact, in a previous study, we have shown that nanoparticles combined with 0.2% of [Cho][Phe] allowed us to incorporate rutin with an AE of 75.6%, which in the absence of the IL was not possible [35,37]. Hence, in this study, we prepared IL-nanoparticles hybrid systems, loaded and unloaded with rutin, using the concentrations of [Cho][Phe] and [Cho][Gly], where cell viability was maintained in Vero cells line. Thus, the [Cho][Phe]–nanoparticle hybrid system was once again prepared but now using a higher percentage of IL (0.3% *v/v*), and for the first time the [Cho][Gly]–nanoparticle hybrid system was produced using 0.2% of the glycinate derivative IL. The physicochemical features and performance of the developed hybrid systems were evaluated. The obtained values for particle size, PdI, and zeta potential are all suitable results for drug delivery and are very similar amongst all the formulations, both for loaded and unloaded and for the two ILs tested. These similarities, as well as the high AE obtained, demonstrate the robustness of the developed delivery systems. Rutin was loaded in each hybrid system at the maximum solubility of this drug in the presence of either IL, namely, 2.49 mg/mL (4100 µM) with 0.3% of [Cho][Phe], and 1.73 mg/mL (2800 µM) with 0.2% of [Cho][Gly]. 

Additionally, the [Cho][Phe]– nanoparticle hybrid system demonstrated that an enhancement of 0.1% of the incorporated IL did not interfere with the hybrid nanosystems stability, but allowed a higher incorporation of the drug and a 10% increase in the AE when compared with the previous prepared hybrid IL–nanocarrier [35]. This shows that the amount of IL impacts drug loading and AE. Also, it should be noted that when using the [Cho][Gly], the newly developed hybrid IL–nanosystem is also stable and that even though this IL is present at a lower percentage (0.2%) compared with [Cho][Phe] (0.3%), the obtained AE is quite similar. The LC values are low for both ILs and similar to those already described in the literature for hybrid IL–nanosystems [37].

Finally, it was also assessed the release of the rutin from the freshly prepared IL–nanoparticle hybrid systems. An initial burst was observed, as expected, probably due to the rutin adsorbed in the surface of the nanoparticles [35,45]. Nonetheless, this burst release of about 45% happened only after 5 h, probably due to a higher affinity of rutin to the IL–nanoparticles hybrid system, followed by sustained release up to 72 h. This release profile is important to enhance the therapeutic effect and reduce the number of administrations required. 

Hence, when considering the low solubility and bioavailability of rutin, and the possible anticancer applicability of this compound, the prepared hybrid IL–nanocarriers may represent a promising strategy to overcome these challenges, presenting a suitable structure and performance and allowing a higher drug loading and controlled drug delivery.

Globally, these results are quite promising and show it would be valuable to continue to study the rutin’s mechanisms of action, to explore its role in cancer progression, and also to assess the efficacy and safety of the rutin loaded IL–nanosystem hybrid systems.

## 5. Conclusions

In summary, this study aimed to investigate the effect, safety, and applicability of rutin as a potential therapeutic agent, in the treatment of human renal cancer and to assess the functionality of ILs to allow the delivery of this poorly soluble drug in a biocompatible manner. 

Our results suggest that rutin displays cytotoxic effects on 786-O human cancer cells and that at 50 µM, it may be safely used against ccRCC since, at this concentration, no significant effect was observed in normal renal cells. Moreover, we also showed that the two choline-amino acid ILs under study can act as functional excipients since their presence at non-toxic concentrations in Vero cells enhanced the drug solubility while allowing the maintenance of the rutin’s activity. Furthermore, ionic liquids–nanoparticle hybrid systems containing rutin were also prepared and our results showed that the ionic liquids were crucial to increasing drug loading and that these IL–nanoparticle hybrid systems may be used as a strategy for rutin delivery, contributing to its applicability as a potential therapeutic agent. These results are promising, showing the potential use of ILs to improve drug delivery and opening a new avenue for further studies to understand the underlying mechanisms of rutin cytotoxicity in order to develop effective and safe hybrid nanocarriers capable of being targeted to the ccRCC.

## Figures and Tables

**Table 1:** Solubility of rutin in water or in water:IL mixtures (99.7:0.3%, *w/w* for [Cho][Phe]; and 99.8:0.2%, *w/w* for [Cho][Gly]); *n* = 3, mean ± SD and *** *p* < 0.001 (one-way ANOVA, Tukey’s test, compared to water solubility).

Solvent	Water:IL Ratio (%)	Rutin Concentration (mg/ mL)
Water	100:0.00	0.19 ± 0.01
Water:[Cho][Phe]	99.7:0.30	2.49 ± 0.12 ***
Water:[Cho][Gly]	99.8:0.20	1.73 ± 0.03 ***

**Table 2:** Association efficiency (AE) and loading capacity (LC) of rutin-loaded ILs-PLGA nanoparticles hybrid systems. Data represented as mean ± SD (*n* = 3).

IL	AE (%)	LC (%)
[Cho][Phe]	84.5 ± 0.3	1.0 ± 0.1
[Cho][Gly]	84.7 ± 0.3	1.1 ± 0.1
